# A Multifunctional Biodegradable Nanocomposite for Cancer Theranostics

**DOI:** 10.1002/advs.201802001

**Published:** 2019-05-21

**Authors:** Jianrong Wu, Gareth R. Williams, Shiwei Niu, Feng Gao, Ranran Tang, Li‐Min Zhu

**Affiliations:** ^1^ College of Chemistry, Chemical Engineering and Biotechnology Donghua University Shanghai 201620 P. R. China; ^2^ UCL School of Pharmacy University College London 29‐39 Brunswick Square London WC1N 1AX UK; ^3^ Department of Ultrasound Shanghai General Hospital Shanghai Jiaotong University School of Medicine Shanghai 200080 P. R. China; ^4^ Women's Hospital of Nanjing Medical University Nanjing Maternity and Child Health Care Hospital Nanjing 210004 China

**Keywords:** biodegradable, chemo‐photothermal therapy, hollow mesoporous organosilica, nanotheranostics, prodrugs, ultrasound imaging

## Abstract

Theranostic formulations, integrating both diagnostic and therapeutic functions into a single platform, hold great potential for precision medicines. In this work, a biodegradable theranostic based on hollow mesoporous organosilica nanoparticles (HMONs) is reported and explored for ultrasound/photoacoustic dual‐modality imaging guided chemo‐photothermal therapy of cancer. The HMONs prepared are endowed with glutathione‐responsive biodegradation behavior by incorporating disulfide bonds into their framework. The nanoparticles are loaded with indocyanine green (ICG) and perfluoropentane (PFP). The former acts as a photothermal agent and the latter can generate bubbles for ultrasound imaging. A paclitaxel prodrug is developed to both serve as a redox‐sensitive gatekeeper controlling ICG release from the HMON pores and a chemotherapeutic. ICG generates mild hyperthermia upon exposure to an 808 nm laser, and this in turn leads to a liquid–gas phase transition of PFP, resulting in the generation of bubbles which can be used for ultrasound imaging. The platform is found to have excellent properties for both ultrasound and photoacoustic imaging. In addition, both in vitro and in vivo results show that the nanoparticles provide potent synergistic chemo‐photothermal therapy. The material developed in this work thus has great potential for exploitation in advanced cancer therapies.

## 1 Introduction

Cancer remains a major challenge to human health globally.^1^, ^2^ Despite an enormous number of potential new cancer therapies having been explored, only limited success has been achieved in translating these to the clinic, in part by dint of the complexity and heterogeneity of tumors.^3^ A potential route to solve this problem is to use theranostics, formulations which contain both a therapeutic element and an imaging agent. Various theranostic materials have been explored for tumor imaging and treatment.^4^, ^5^ Particular interest has been placed on the use of nanomaterials in this regard, as a result of their ability to enter and accumulate in tumor cells; nanoscale theranostics can thus significantly improve diagnostic or/and therapeutic efficacy.^6^

A wide range of organic and inorganic nanomaterial‐based theranostic systems have been explored, including mesoporous organosilica nanoparticles (MONs).^7^, ^8^, ^9^ The latter are similar to mesoporous silicas, but rather than comprising pure SiO_2_ contain organic functional groups within the silica framework. MONs are promising carriers for therapeutic/diagnostic modalities because particles can be made with well‐defined morphologies, large surface areas, and good biocompatibility; further, by varying the organic groups present, the MON degradation rate and location can be controlled.^10^, ^11^ Various organic moieties can be incorporated into MONs, and since these are intrinsic components of the framework they do not block the pore channels. 12, 13

Given their organic–inorganic hybrid structure, MONs have greater potential than pure SiO_2_‐based nanocarriers for drug delivery and theranostic platforms.^14^, ^15^, ^16^, ^17^ For instance, Huang et al. fabricated hollow MONs with a physiologically active disulfide bond (—S—S—) incorporated into the silica framework.^15^ They successfully employed this material as a carrier for doxorubicin, using the vulnerability of the —S—S— linkage to the reducing environment typically found in a tumor to target drug delivery through disintegration of the framework.^16^ In other work, Chen and co‐workers reported a nanotheranostic based on CuS‐modified MONs for tumor‐specific perfluoropentane delivery and multimodality imaging.^18^

Although there has recently been great progress in research into MONs for biomedical applications, most of the systems explored to date still have critical problems, such as a lack of an appropriate “gatekeeper” to cap the pore entrances of the nanoparticles. This is important to avoid premature leakage of the payloads into the blood circulation before the target is reached.^19^ Therefore, it is highly desirable to design new platforms that are capped by agents with negligible systemic toxicity and which could respond to endogenous triggers to provide efficient cancer treatment.

A wide range of diagnostic tools are employed in the clinic, with noninvasive probes being preferred wherever possible. Ultrasound (US) is one such technique, and has advantages of bedside availability and allowing real‐time imaging.^20^, ^21^ A number of contrast agents comprising gas‐filled microbubbles can be used for US imaging. However, most of the bubbles are micrometer‐sized and their lifetime in vivo is short (<20 min).^22^ Since micrometer‐sized bubbles cannot enter a tumor, they need to be generated in situ, and enhancement of bubble stability would also be highly beneficial for monitoring treatment. Nanoscale gas‐generating platforms offer one route to tackle these issues, particularly if the slightly acidic tumor environment can be exploited to trigger gas release.^23^ Even so, US imaging is not suitable for the early diagnosis of cancer due to its inherently poor contrast and limited spatial resolution. Photoacoustic (PA) imaging, a hybrid imaging method combining optical and ultrasound properties, allows these limitations to be overcome.^7^ PA imaging can improve both the resolution and depth of the US approach.^22^ Therefore, the combination of PA with US imaging would permit early diagnosis and accurate monitoring of treatment in real time.

To develop US or PA imaging systems, nanoscale platforms can be used to encapsulate stable species (e.g., CaCO_3_,^24^ perfluoropentane (PFP),^25^ or perfluorohexane (PFH)^26^). These can enter tumors and then induce the generation of nanobubbles. The nanobubbles will subsequently grow to micrometer‐size, providing enhanced US contrast. Bubble generation typically occurs when a liquid cargo is heated to a temperature exceeding its boiling point.^27^, ^28^ Mild hyperthermia induced by photothermal conversion agents under near‐infrared (NIR) irradiation is one route that can be used to vaporize the liquid.^18^ For example, Shi and coworkers established a multifunctional theranostic nanoplatform based on Au nanoparticle‐coated hollow mesoporous silica loaded with PFH, and showed this to have applications for multimodal US/computerized tomography/photoacoustic/thermal imaging of tumors.^26^

**Scheme 1:**
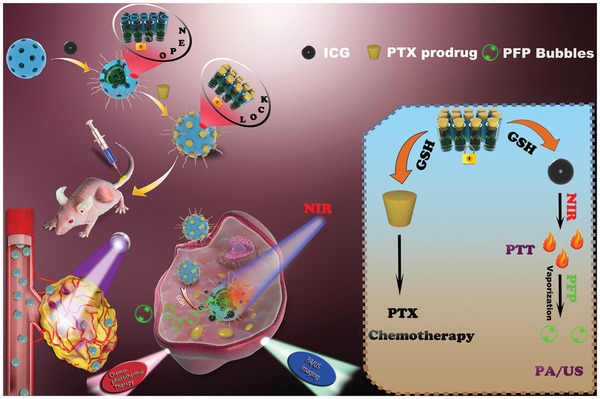
Schematic illustration of the construction of ICG/PFP@HMOP‐PEG for PA/US imaging and chemo‐photothermal therapy.

Despite recent progress, there is still much work to do in the development of biodegradable nanoscale theranostic platforms. In this work, we report a novel material with tumor‐sensitive biodegradability which allows high‐quality US/PA imaging combined with synergistic chemo‐photothermal therapy. Hollow mesoporous organosilica nanoparticles (HMONs) with sub‐75 nm particle size were constructed and loaded with PFP (which boils at 29 °C)^29^ and the photothermal agent indocyanine green (ICG). Further, a disulfide‐containing paclitaxel (PTX) prodrug was incorporated at the nanoparticle surface, both providing a drug payload and acting as gatekeeper to the HMON pores. The prodrug prevents the premature release of the encapsulated species, and also endows the material with redox‐responsive ICG release properties. Finally, polyethylene glycol (PEG) was introduced to the surface to improve colloidal stability (**Scheme** 1), yielding particles termed ICG/PFP@HMOP‐PEG. The resultant formulation has significant potential for combined ultrasound‐photoacoustic imaging guided tumor therapy, and ultimately could yield major benefits for patients.

## 2 Results and Discussion

### 2.1 Design, Fabrication, and Characterization of ICG/PFP@HMOP‐PEG

**Figure 1:**
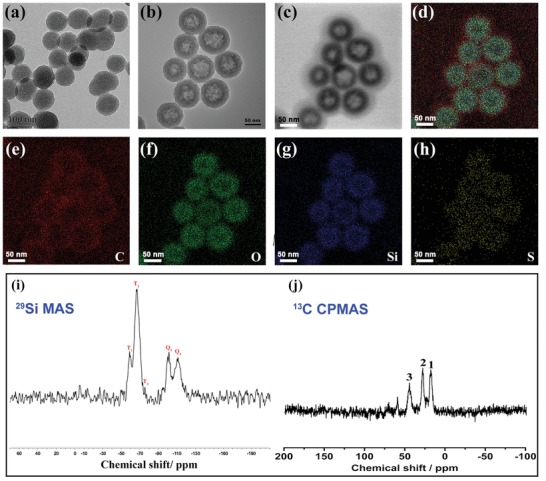
Characterizing data on the HMONs, including transmission electron microscopy (TEM) images of a) SiO_2_@MONs and b) HMONs. c) A bright‐field scanning transmission electron microscopy image of the HMONs with d) total elemental mapping and individual maps for e) C, f) O, g) Si, and h) S. Scale bar: 50 nm. i) ^29^Si and j) ^13^C NMR spectra of the as‐synthesized HMONs.

The incorporation of disulfide bonds into silica frameworks can endow them with tumor sensitive biodegradability,^30^, ^31^, ^32^ owing to the reducing environment of the latter. Disulfide bond‐containing HMONs were thus synthesized by an ammonia‐assisted selective etching method.^15^ This resulted in core/shell SiO_2_@MONs (**Figure** 1a), with triethanolamine used to ensure they had a small particle size.^33^ The SiO_2_ core was removed by etching in an ammonia solution, resulting in hollow HMONs of around 75 nm in size (Figure 1b). Elemental mapping (Figure 1c–h) shows the presence and uniform distribution of C, S, Si, and O in the HMONs. Energy‐dispersive X‐ray spectroscopy analysis further confirms the presence of these elements in the structure (Figure S1, Supporting Information).

The structure of the HMONs was analyzed by ^29^Si magic‐angle spinning (MAS) and ^13^C cross‐polarization MAS (CPMAS) solid‐state NMR. Both Q and T sites were observed in the ^29^Si spectrum (Figure 1i), indicating the presence of a hybrid silsesquioxane framework.^34^ Characteristic resonances at 17.4, 26.3, and 45.6 ppm in the ^13^C CPMAS NMR spectrum can be assigned to the carbon species in the —Si—^1^CH_2_—^2^CH_2_—^3^CH_2_—S—S—^3^CH_2_—^2^CH_2_—^1^CH_2_—Si— units (Figure 1j). Raman spectroscopy was used to confirm the presence of disulfide bonds within the HMONs; distinct stretching vibrations at 487 and 506 cm^−1^ are seen in the spectrum, confirming the existence of the —S—S— bond (Figure S2, Supporting Information).^35^ The porosity of the HMONs was assessed through N_2_ adsorption–desorption isotherms (Figure S3, Supporting Information). These reveal the presence of a well‐defined mesoporous structure, with a surface area of 869.5 m^2^ g^−1^ and uniform pore size of 4.14 nm, (Figure S3, Supporting Information). This makes the HMONs promising for the delivery of a range of payloads.

**Figure 2:**
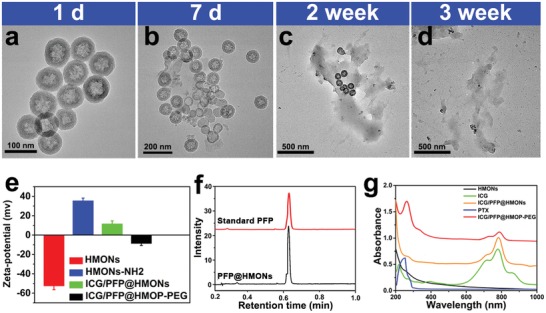
TEM images of HMONs dispersed in 10 × 10^−3^ m GSH aqueous solution for a) 1 days, b) 7 days, c) 2 weeks, and d) 3 weeks. e) The zeta potentials of HMONs, HMONs‐NH_2_, ICG/PFP@HMONs, and ICG/PFP@HMOP‐PEG. f) Gas chromatogram of PFP and PFP from PFP@HMONs. g) UV–vis–NIR spectra of HMONs, free ICG, ICG/PFP@HMONs, and ICG/PFP@HMOP‐PEG.

The disulfide bonds in the HMON framework should break in response to a reducing microenvironment,^36^ and therefore their biodegradation behavior was evaluated in simulated body fluids containing glutathione (10 × 10^−3^ m) and monitored by TEM. A time‐dependent behavior was observed (**Figure** 2a–d). The HMONs were degraded gradually over a period of approximately two weeks, after which almost no intact particles remain.

Subsequently, the HMONs were modified with 3‐aminopropyltriethoxysilane (APTES) to introduce amino groups (forming HMONs‐NH_2_). This was done following a previously reported protocol.^37^ The zeta potential of the initial HMONs was ≈−52.5 mV, but this increased to +35.5 mV after modification with APTES (Figure 2e). A slight decrease in specific surface area (675 m^2^ g^−1^) and average pore size (3.82 nm) compared to the blank HMONs were observed (Figure S3, Supporting Information), but amino functionalization did not alter the mesoporous structure.

Temperature‐sensitive PFP molecules were next loaded into the core of the HMONs using a mild infusion method.^27^ When free PFP is added to phosphate buffered saline (PBS), there is clear phase separation because PFP is hydrophobic (Figure S4a, Supporting Information). In contrast, the hydrophilic PFP‐loaded HMONs (PFP@HMONs) can be well dispersed in PBS (Figure S4b, Supporting Information), indicating that PFP could be loaded into the HMONs. This was verified by gas chromatography (Figure 2f); the observed retention time agrees well with the PFP standard.^23^ The near‐infrared fluorescence dye ICG was then additionally loaded into the PFP@HMONs (yielding ICG/PFP@HMONs). UV–vis–NIR absorption spectra of the resultant composites (Figure 2g) show the characteristic absorbance peaks of ICG in the NIR region, indicating that ICG was successfully loaded into the particles. The loading capacity of ICG was 46.1% (w/w), as determined by UV–vis spectroscopy.

The ICG/PFP@HMONs nanoparticles were next gated with a PTX prodrug to form ICG/PFP@HMOP. PTX was first reacted with 3,3′‐dithiodipropionic acid to obtain the prodrug PTX‐SS‐COOH (Figure S5, Supporting Information), and successful synthesis confirmed by ^1^H NMR (Figure S6, Supporting Information). The characteristic 2′‐hydroxyl group of PTX displays a shift from 4.75 to 6.5 ppm in PTX‐SS‐COOH, and new peaks arise at 2.6–2.8 ppm, confirming the formation of the prodrug. The content of PTX in the prodrug was assessed by UV spectroscopy (λ = 227 nm).^38^ The PTX content of PTX‐SS‐COOH was ≈94.65 wt%, close to the theoretical content (97.2 wt%). PTX‐SS‐COOH was reacted with the ICG/PFP@HMONs via the formation of amide bonds between carboxyl and amino groups on the pore outlets. After PEGylation, the final nanoplatform (ICG/PFP@HMOP‐PEG) was obtained. The PTX content in the ICG/PFP@HMOP‐PEG nanoparticles was calculated to be 13.8% wt%.

The anchoring of the PTX prodrug was confirmed by the change of zeta potential from +11.6 to −8.7 mV, and a distinct decrease in pore size and surface area (Figure 2b; Figure S3, Supporting Information). Successful functionalization with the prodrug gatekeeper was further investigated by FTIR and UV–vis–NIR spectroscopy. The former (Figure S7, Supporting Information) shows the characteristic vibration band of amide groups at 1650 cm^−1^, and the distinctive absorption peak of PTX is visible in the UV spectrum (Figure 2g). All these results confirm the successful conjugation of the PTX prodrug onto the pore outlet of HMONs.

ICG/PFP@HMOP‐PEG can be well‐dispersed in a range of different media (water, PBS, and DMEM), and exhibited a hydrodynamic size of 115 ± 4 nm in PBS, with a PDI of 0.274 (Figure S8, Supporting Information). Moreover, the hydrodynamic size of the nanoparticles showed no significant change after aging in PBS for 7 days, only a slight tendency for size to increase, indicating ICG/PFP@HMOP‐PEG should be stable upon storage (Figure S9, Supporting Information).

### 2.2 Glutathione (GSH)‐Dependent Drug Release and NIR‐Triggered PFP Microbubble Generation

**Figure 3:**
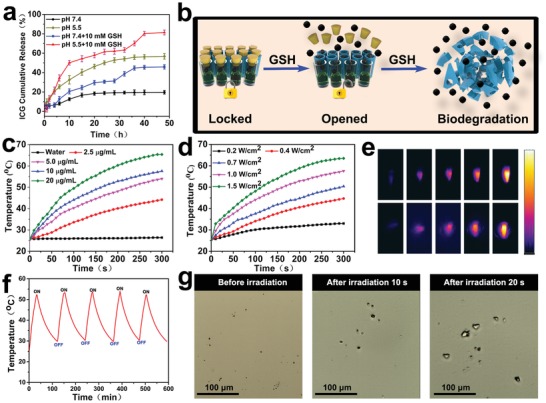
a) ICG release from ICG/PFP@HMOP‐PEG. b) Schematic illustration of GSH‐responsive drug release from ICG/PFP@HMOP‐PEG. c) Temperature increase profiles for ICG/PFP@HMOP‐PEG suspensions at different concentrations upon NIR laser irradiation (808 nm, 1.0 W cm^−2^). d) Temperature increase profiles for ICG/PFP@HMOP‐PEG (5.0 µg mL^−1^) upon NIR laser irradiation with different power densities. e) Photothermal images of ICG/PFP@HMOP‐PEG after NIR laser irradiation for 5 min, with different particle concentrations (top) and power densities (bottom). f) Photothermal stability of ICG/PFP@HMOP‐PEG over five on/off cycles of NIR laser irradiation. g) Optical microscopy images of ICG/PFP@HMOP‐PEG suspensions in water before and after NIR laser irradiation.

Gatekeeper molecules need to be both biocompatible and biosensitive.^19^, ^39^ Here, we used a PTX prodrug as a GSH‐sensitive gatekeeper. To investigate the gating effect, ICG/PFP@HMONs‐PEG was also prepared as a control and ICG release investigated at different pH (pH 5.5 and pH 7.4) in the presence or absence of GSH (10 × 10^−3^ m). A significant burst release was observed for ICG/PFP@HMONs‐PEG with >78% release of ICG reached within 6 h (Figure S10, Supporting Information). By contrast, less than 20% of ICG is released from ICG/PFP@HMOP‐PEG at pH 7.4 over 48 h (**Figure** 3a), confirming that the anchored prodrug prevents the premature release of ICG.

As shown in Figure 3a, the presence of GSH clearly promotes ICG release, which reaches 81.4% at pH 5.5 and 45.9% at pH 7.4 after 48 h when GSH is added. Moreover, multistage release behavior was obtained. Approximately 50% ICG release was observed in the first 4 h (GSH 10 × 10^−3^ m, pH 5.5), after which slower release ensues. In the last 16 h, a significant increase in ICG release is observed. This behavior may be due to the initial cleavage of disulfide bonds and consequent elimination of the PTX prodrug gatekeeper, and then the subsequent biodegradation of the HMON framework (Figure 3b). Considering that high GSH concentrations are present in cancer cells,^30^ the biodegradation behavior of the HMONs allows us to achieve tumor‐targeted release of drug payloads (Figure 3b). PTX release from ICG/PFP@HMOP‐PEG was also explored (Figure S11, Supporting Information). PTX release is rapid, and levels off at around 60% after 10 h in 10 × 10^−3^ m GSH solution.

As ICG displays strong absorption in the NIR region, efficient light‐to‐heat conversion should occur. As expected, the ICG/PFP@HMOP‐PEG material displays strong photothermal effects (Figure 3c–e) and both concentration (Figure 3c) and laser power density (Figure 3d,e) dependent temperature increases were observed. The imaging data in Figure 3e show the ICG/PFP@HMOP‐PEG to have potent thermal imaging properties, and thus the generation of mild hyperthermia and gasification of PFP can be achieved by adjusting the concentration or laser power density. The photostability of ICG/PFP@HMOP‐PEG and free ICG were measured; the former has excellent photothermal stability over 5 cycles of irradiation (Figure 3f), while for the latter a significant loss in performance was noted (Figure S12, Supporting Information).

Encouraged by the light‐to‐heat conversion properties of ICG, we next tested the liquid–gas phase transformation of the PFP cargo when ICG/PFP@HMOP‐PEG was exposed to an 808 nm NIR laser. The boiling point of PFP will rise from 29 to 45 °C in the blood circulation due to the effect of blood pressure,^40^ so a laser power density of 1.0 W cm^−2^ was used to generate a temperature of 55 °C and induce the generation of microbubbles. Optical microscopy images (Figure 3g) show increasing numbers of microbubbles were observed with prolonged laser irradiation time, and that ICG/PFP@HMOP‐PEG did not produce any bubbles without laser irradiation (Figure 3g). This confirmed that NIR induces a phase transition of PFP, and the resultant microbubbles could be used for ultrasound imaging.

### 2.3 In Vitro Imaging Performance

**Figure 4:**
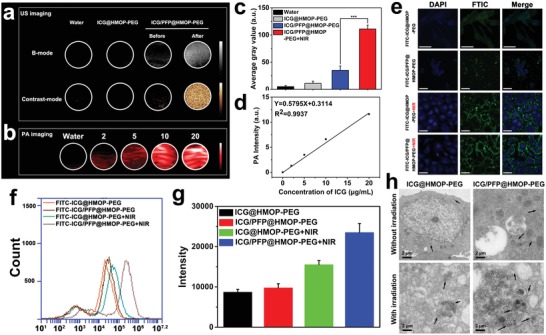
a) US images in B‐mode and contrast mode of water, ICG@HMOP‐PEG, and ICG/PFP@HMOP‐PEG before and after NIR irradiation. b) PA images of water and ICG/PFP@HMOP‐PEG suspensions at various concentrations (2, 5, 10, and 20 mg mL^−1^) after NIR irradiation. c) Quantitative analysis of the corresponding average gray values in (a). d) Quantitative analysis of the relationship between the PA signal intensity and the concentration of ICG in (b). e) Confocal fluorescence images of MDA‐MB‐231 cells incubated with FITC‐conjugated ICG@HMOP‐PEG and ICG/PFP@HMOP‐PEG, without or with NIR laser irradiation. Scale bar: 50 µm. f) Flow cytometry analysis and g) FITC intensity values for the uptake of FITC ‐labeled ICG@HMOP‐PEG and ICG/PFP@HMOP‐PEG. h) Bio‐TEM images of MDA‐MB‐231 cells incubated with ICG@HMOP‐PEG and ICG/PFP@HMOP‐PEG with or without laser irradiation.

The US imaging properties of ICG/PFP@HMOP‐PEG were investigated using B‐mode and contrast‐intensified US (CEUS) with or without laser irradiation (808 nm, 1.0 W cm^−2^). As shown in **Figure** 4a, weak US signals and no obvious changes with laser exposure were observed for water and ICG@HMOP‐PEG in both the B‐mode and CEUS images. By contrast, the signal intensities of the ICG/PFP@HMOP‐PEG particles are significantly different before and after laser exposure in both B‐mode and contrast mode, because of the ICG‐mediated liquid–gas phase transition of PFP. PA imaging is an attractive technique permitting high spatial resolution and deep tissue penetration,^23^, ^41^, ^42^ and thus the PA imaging performance of ICG/PFP@HMOP‐PEG at different ICG concentrations was also explored with the excitation wavelength from 700 to 950 nm and a 10 nm interval. A concentration‐dependent PA signal was observed for ICG/PFP@HMOP‐PEG (Figure 4b). Both the US and PA signals can be quantified. The average US gray value of ICG/PFP@HMOP‐PEG under NIR irradiation is almost three times higher than that before laser irradiation (Figure 4c). This confirmed that ICG/PFP@HMOP‐PEG has the potential for enhancing US imaging. Quantitative analysis also reveals a highly linear correlation between the PA signal intensity and the ICG concentration (Figure 4d). The ICG/PFP@HMOP‐PEG system hence has outstanding imaging ability in both US and PA imaging.

### 2.4 In Vitro Bubble‐Enhanced Cellular Uptake

It has been reported that bubbles can enhance the cellular uptake of nanoparticles, due to permeable defects in cell membranes being generated by PFP microbubbles.^43^ The in vitro cellular uptake efficiency of ICG/PFP@HMOP‐PEG was studied via confocal laser scanning microscopy (CLSM) and flow cytometry. Since IGC cannot be detected by CLSM or flow cytometry, FITC‐labeled versions of ICG@HMOP‐PEG or ICG/PFP@HMOP‐PEG were prepared. MDA‐MB‐231 cancer cells were incubated with FITC‐ICG@HMOP‐PEG or FITC‐ICG/PFP@HMOP‐PEG, with or without laser irradiation, and the fluorescence of FITC in the cells was measured (Figure 4e).

The observation of weak green fluorescence for both FITC‐ICG@HMOP‐PEG and FITC‐ICG/PFP@HMOP‐PEG confirms a relatively low degree of cellular uptake. Uptake is enhanced when FITC‐ICG@HMOP‐PEG is exposed to NIR irradiation, since the resultant mild hyperthermia induces minor disruptions to the cell membrane.^44^ For cells incubated with FITC‐ICG/PFP@HMOP‐PEG and NIR‐irradiated, the FITC signal is stronger again. Here, the temperature increase can both directly cause disruption to the membrane and also generate PFP bubbles, which will cause further disruption. Quantitative analysis by flow cytometry (Figure 4f) confirmed these findings: FITC fluorescence with FITC‐ICG/PFP@HMOP‐PEG was 1.87‐fold higher than with FITC‐ICG@HMOP‐PEG at the same power density laser irradiation (Figure 4f). Also, the mild hyperthermia‐induced generation of PFP enhances the tumor cell uptake may be ascribed to the PFP bubbles driving more nanoparticles into the cytoplasm.^18^

The uptake of ICG@HMOP‐PEG and ICG/PFP@HMOP‐PEG with or without laser irradiation was further evaluated at MDA‐MB‐231 cell level by direct observation of the microstructure evolution intracellularly via bio‐TEM (Figure 4h). After laser irradiation, greater numbers of ICG/PFP@HMOP‐PEG nanoparticles are present within the cells than is the case with ICG@HMOP‐PEG. Only a few nanoparticles can be found with either material without laser irradiation, which is in agreement with the flow cytometry and CLSM findings. Thus, the generation of PFP bubbles clearly facilitates cellular uptake of the nanoparticles.

### 2.5 In Vitro Therapeutic Effects

**Figure 5:**
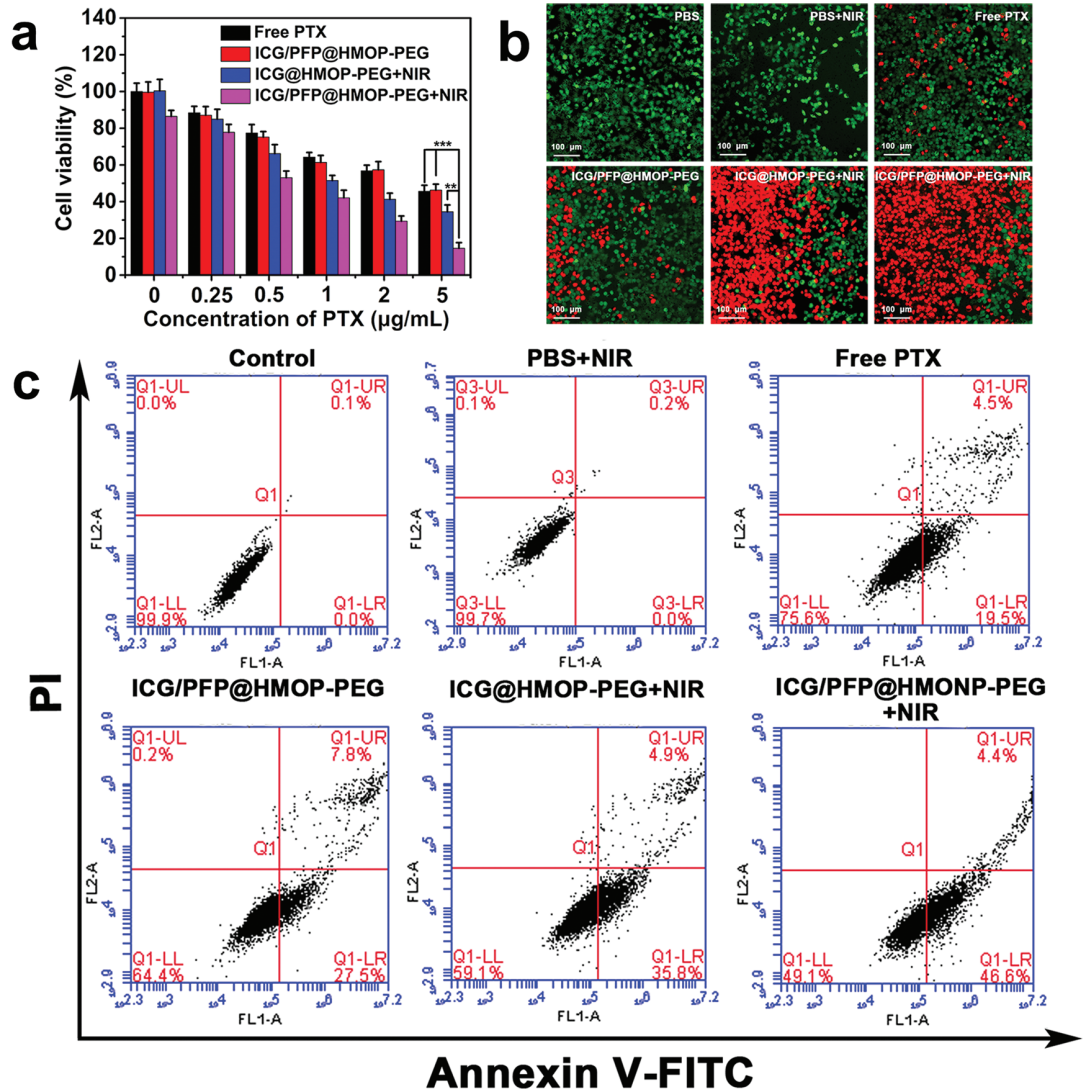
a) In vitro viability of MDA‐MB‐231 cells. **P < 0.01, ***P < 0.001. b) Calcein‐AM/PI staining images of MDA‐MB‐231 cells after different treatments (scale bar = 100 µm). c) Flow cytometry results for Annexin V‐FITC and PI costained MDA‐MB‐231 cells. UL: necrotic cells; LL: living cells; UR: late apoptotic cells; UL: early apoptotic cells.

The in vitro therapeutic efficacy of ICG/PFP@HMOP‐PEG was evaluated using a Cell Counting Kit‐8 assay. The HMON and PFP@HMONs‐PEG nanoparticles exhibited negligible toxicity to cells, even at high concentrations (>90% viability at 200 µg mL^−1^; Figure S13, Supporting Information), showing them to have high biocompatibility. After ICG loading and prodrug capping, the cytotoxicity of the different formulations, including free PTX, the PTX prodrug, and ICG/PFP@HMOP‐PEG was examined. Similar dose‐dependent cytotoxicity was displayed for PTX and ICG/PFP@HMOP‐PEG (**Figure** 5a), confirming that formation of the prodrug and its attachment to the nanocarrier do not compromise the therapeutic efficacy of PTX.

The cell viability after treatment with ICG/PFP@HMOP‐PEG and NIR irradiation decreased dramatically to 14.6% at a PTX concentration of 5 µg mL^−1^, which is significantly lower than ICG@HMOP‐PEG due to PFP bubble‐induced cellular uptake. A calcein AM/propidium iodide (PI) stain was performed to verify these findings (Figure 5b). Cells treated with ICG/PFP@HMOP‐PEG and NIR showed intense red fluorescence, confirming the presence of numerous dead cells, many more than were noted with other treatments.

Cellular apoptosis was quantified by flow cytometry. As shown in Figure 5c, cells treated with ICG/PFP@HMOP‐PEG under laser irradiation showed greatly increased apoptosis in comparison to cells treated with free PTX or ICG/PFP@HMOP‐PEG in the absence of light irradiation. Fewer cells were apoptotic/necrotic after treatment with ICG@HMOP‐PEG under laser irradiation, which may be attributed to the lack of PFP.

To further investigate the effect of ICG/PFP@HMOP‐PEG under laser irradiation on MDA‐MB‐231 cell apoptosis, RT‐qPCR was used to detect the mRNA expression levels of several important apoptosis‐related genes (Bcl‐2, Bax, and Caspase‐3). It can be observed that PTX, ICG/PFP@HMOP‐PEG and ICG@HMOP‐PEG under laser irradiation significantly inhibit the expression of *Bcl‐2* mRNA and promote the process of cancer cell apoptosis (Figure S14a, Supporting Information). The downregulation effect of ICG/PFP@HMOP‐PEG under laser irradiation was greater than that of the other formulations (*P* < 0.01). *Bax* (an inhibitor of Bcl‐2), and Caspase‐3 genes are activated in apoptotic cells both by extrinsic and intrinsic pathways, and were found to be upregulated in MDA‐MB‐231 cells after the different treatments (Figure S14b,c, Supporting Information). Cells treated with ICG/PFP@HMOP‐PEG under laser irradiation exhibited the highest mRNA expression levels for these pro‐apoptosis genes. These results together demonstrate that ICG/PFP@HMOP‐PEG possesses synergistic chemo‐photothermal properties and has great potential in anticancer therapy.

### 2.6 In Vivo Dual‐Modality Imaging

**Figure 6:**
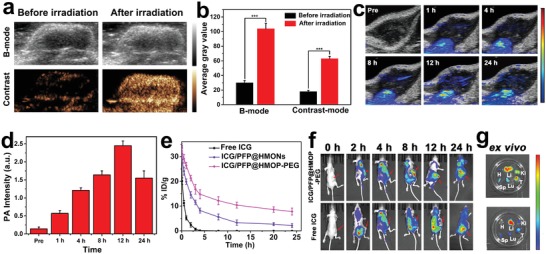
Data for in vivo experiments performed with ICG/PFP@HMOP‐PEG in MDA‐MB‐231 tumor‐bearing nude mice. a) In vivo US images and b) the corresponding gray values of the tumor obtained 24 h after injection (***P < 0.001). c) In vivo PA images and d) the corresponding PA intensity values of the tumor tissues as a function of time. e) The pharmacokinetics of free ICG, IGC/PFP@HMONs, and ICG/PFP@HMOP‐PEG, expressed as injected dose per gram of tissue (%ID/g). f) In vivo fluorescence images taken at different time points (arrows denote the tumor position). g) Ex vivo fluorescence images of different organs and the tumor (H, Li, Sp, Lu, Ki, and T denote the heart, liver, spleen, lung, kidney, and tumor, respectively).

In vivo imaging was carried out for MDA‐MB‐231‐tumor‐bearing mice after i.v. injection with ICG/PFP@HMOP‐PEG (3 mg mL^−1^, in terms of ICG). As shown in **Figure** 6a, there was a faint signal in the tumor 24 h after injection of ICG/PFP@HMOP‐PEG without NIR laser irradiation. This is because the phase change temperature of PFP in vivo is higher than the physiological temperature (37 °C) due to the blood/intratumor pressure. The US signal in B‐mode was significantly enhanced after mild NIR laser irradiation (1.0 W cm^−2^, 5 min). This treatment will raise the tumor temperature to be around 50 °C, leading to generation of PFP nanobubbles and their coalescence into microbubbles. Similar results are noted for CEUS measurements. The average gray value after laser irradiation is significantly higher than the preinjection value under either B‐mode or CEUS mode (Figure 6b).

PA imaging was also performed (Figure 6c). The PA signals from the tumor region increase with time, and reach a maximum at 12 h post‐injection, demonstrating the tumor retention of ICG/PFP@HMOP‐PEG. A strong PA signal was still visible at 24 h, but the intensity had declined from that seen at 12 h. This arises because of the biodegradation of a portion of the ICG/PFP@HMOP‐PEG. The quantitative PA signal was determined, and the results follow the trend seen in the images (Figure 6d). Clearly, ICG/PFP@HMOP‐PEG can accumulate in the tumor and act as a contrast agent for both US and PA imaging.

### 2.7 In Vivo Pharmacokinetics and Biodistribution

The in vivo pharmacokinetics of ICG/PFP@HMOP‐PEG were monitored by measuring the fluorescence of ICG in the blood of the mice. ICG/PFP@HMOP‐PEG displayed an enhanced blood circulation half‐life compared with ICG/PFP@HMONs and free ICG (Figure 6e). The area under the curve (AUC) of ICG/PFP@HMOP‐PEG was distinctly greater than the AUC for the other two formulations (Table S1, Supporting Information). These results confirmed that ICG/PFP@HMOP‐PEG could effectively extend the blood circulation time of ICG due to the PTX prodrug capping and PEGylation. These results are highly favorable for increasing tumor accumulation.^39^

The in vivo distribution and tumor accumulation of ICG/PFP@HMOP‐PEG in MDA‐MB‐231 tumor‐bearing mice were next evaluated by tracking the fluorescence of ICG using an in vivo NIR fluorescent imaging system. As depicted in Figure 6f, ICG fluorescence was observed throughout the whole body 2 h after intravenous injection of ICG/PFP@HMOP‐PEG. The fluorescence of ICG gradually increases, with the greatest signal visible at the tumor site after 8 h. A significant intensity is maintained at 12 and 24 h, demonstrating the tumor accumulation and retention of ICG/PFP@HMOP‐PEG. By contrast, for mice given a solution of ICG the fluorescence intensity in the tumors increased up to 8 h postinjection, but then decreased owing to the short circulation time of ICG. This high tumor accumulation of ICG/PFP@HMOP‐PEG can be attributed to the widely reported enhanced permeability and retention (EPR) effect.^7^, ^10^, ^12^, ^18^

After 24 h, the mice were euthanized and ex vivo fluorescence imaging was performed on the major organs (heart, liver, spleen, lung, and kidney) and tumor tissue (Figure 6g). Mice treated with ICG/PFP@HMOP‐PEG exhibited much stronger ICG fluorescence in the tumor than in the major organs. Distinct fluorescence is present in the liver and kidneys, as would be expected because the platform begins to degrade and be excreted over this time frame. The accumulation of nanoparticles in the liver can be attributed to absorption by the mononuclear phagocyte system; those accumulated in the kidney are likely to arise from renal excretion.^14^, ^45^ Quantitative analysis of the ICG fluorescence intensity distribution in the organs and tumor is shown in Figure S15 of the Supporting Information, and is in agreement with the ex vivo fluorescence imaging. ICG accumulation in the tumor is significantly greater with the nanoplatform than with free ICG. It is thus clear that ICG/PFP@HMOP‐PEG enjoys a long retention time in the blood stream, and extensive tumor uptake via the EPR effect.

### 2.8 In Vivo Antitumor Activity and Biosafety

**Figure 7:**
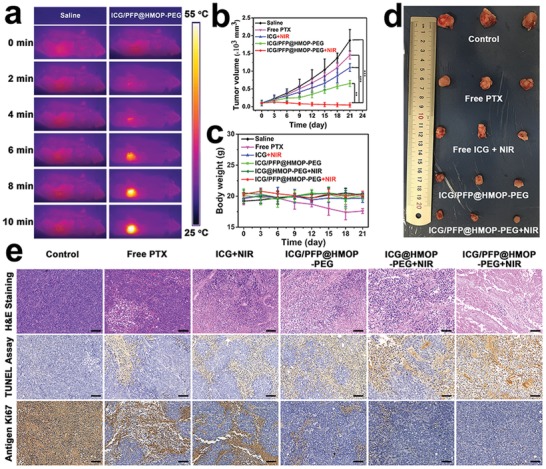
In vivo anticancer properties of ICG/PFP@HMOP‐PEG after i.v. injection into MDA‐MB‐231 tumor‐bearing nude mice. a) In vivo photothermal images under 808 nm laser irradiation. b) Relative tumor volume and c) body weight changes with time. d) Photographs of the tumors excised after 21 d. e) H&E, TUNEL, and Ki‐67 staining of tumor sections (scale bars: 50 µm).

To evaluate the in vivo anticancer properties of the formulations, mice were randomly divided into five groups (*n* = 5 per group) and treated as follows: saline, free PTX, ICG/PFP@HMOP‐PEG, free ICG+NIR, ICG@HMOP‐PEG+NIR or ICG/PFP@HMOP‐PEG+NIR. For the groups receiving NIR, mice were exposed to an 808 nm NIR laser (1.0 W cm^−2^) for 10 min at 8 h postinjection. Thermal images were recorded using an IR thermal camera (**Figure** 7a). The temperature of the tumor in mice treated with ICG/PFP@HMOP‐PEG+NIR increased to 52.4 °C after laser irradiation while saline‐treated mice display the normal physical temperature (≈35 °C) at the tumor site (Figure S16, Supporting Information), confirming the high photothermal conversion efficiency of ICG/PFP@HMOP‐PEG.

The tumor volume and body weights were monitored throughout a 21‐day treatment period. As is clear from Figure 7b, the administration of free PTX, and ICG/PFP@HMOP‐PEG partially inhibit tumor growth due to the chemotherapeutic properties of PTX. In contrast, the volume of the tumors in mice treated with saline sharply increased during the treatment period. Mice given free ICG and NIR showed only a small reduction in tumor volume when compared to the saline group. This is because of the limited accumulation of free ICG in the tumor. The mice in the ICG/PFP@HMOP‐PEG+NIR group showed the most profound inhibition of tumor growth, with the PFP‐containing NPs being more potent than the ICG@HMOP‐PEG+NIR group (Figure S17, Supporting Information) owing to the mild hyperthermia‐induced liquid–gas transformation of PFP enhancing uptake.^18^ Considering the body weight data (Figure 7c), it is evident that mice injected with free PTX showed a distinct drop in body weight with time, a result of the systemic toxicity of PTX.^46^ However, no significant body weight changes were observed with any of the other treatments (Figure 7c), indicating the biocompatibility of the materials. Digital photos of the excised tumors (Figure 7d) confirm that the ICG/PFP@HMOP‐PEG+NIR group possessed the smallest tumor size.

Tumor sections were taken from the mice after sacrifice and stained with H&E, TUNEL, and the Ki‐67 antibody. In H&E staining assays (Figure 7e), significant apoptosis/necrosis regions are visible in the tumors from the ICG/PFP@HMOP‐PEG+NIR group, while the other treatment groups appear much more similar to the negative control. The presence of apoptotic cells (stained brown in the TUNEL images) is most distinct in sections from the ICG/PFP@HMOP‐PEG+NIR group, and is significantly higher than in the other groups. Immunochemical staining with Ki‐67 on tumor sections confirms the TUNEL and H&E data. The ICG/PFP@HMOP‐PEG+NIR group exhibited the lowest level of cellular proliferation (the fewest brown nuclei) of all the treatments, further indicating the synergistic effects induced by combined chemotherapy and PTT.

The biocompatibility and biosafety of the ICG/PFP@HMOP‐PEG platform were evaluated by histology and serum biochemistry assays. H&E‐stained images of major organ sections (heart, liver, spleen, lung, and kidney) displayed no obvious damage after treatment with ICG/PFP@HMOP‐PEG and ICG/PFP@HMOP‐PEG+NIR (Figure S18, Supporting Information), indicating the nanoparticles do not cause any significant organ damage. Similarly, when healthy mice were treated with ICG/PFP@HMOP‐PEG+NIR there were no differences in any serum biomarkers between the treatment and control group, and all parameters lay in the normal range (Figure S19, Supporting Information). This confirms ICG/PFP@HMOP‐PEG to have a good hepatic and kidney safety profile.

The ICG/PFP@HMOP‐PEG gas generation system developed in this study has great potential for use as a nanoscale theranostic platform. Upon NIR laser irradiation, PFP bubbles are generated, as a result of mild hyperthermia (Figure 3g). These not only enhance the tumor cell uptake of the nanoparticles (Figure 4e–h), but also serve as an ultrasound imaging agent (Figure 4a). The hollow mesoporous organosilica nanoparticles comprising the carrier have intrinsic tumor microenvironment responsive biodegradability (Figure 2a–d), and excellent biocompatibility/biosafety (Figures S18 and S19, Supporting Information).^14^, ^15^

HMONs and other mesoporous silica systems have been constructed for the delivery of PFP and perfluorohexane in several recent studies,^18^, ^27^ and found to act as potent bubble generators. However, the previously reported platforms lack a gatekeeper, which leads to problems with the premature release of PFP.^19^ Here, a PTX prodrug was employed as a GSH‐sensitive “gatekeeper”, both to minimize the potential risks of adding auxiliary capping agents and act as a chemotherapeutic. In situ gas generation has been reported to act as a deep‐penetrating drug delivery system for improved chemotherapy,^47^ and the prodrug‐capping strategy employed in this work also has potential for codelivery of other drugs or biomacromolecules (such as DNA, enzymes, and proteins). Many nanomaterials have been developed to serve as carriers for ICG delivery. The HMONs used in this work show a high loading of ICG (46.1%, w/w), which is significantly higher than previously reported ICG carriers such as MoS_2_ (5.1%),^48^ liposomes (7.41%),^49^ CuS@SiO_2_ (12.3%),^50^ and mesoporous silica (6.68%).^37^ This, along with mild hyperthermia‐induced bubble generation and enhanced celluar uptake, endows the ICG/PFP@HMOP‐PEG material with great potential for dual‐modality imaging guided chemo‐photothermal therapy while obviating detrimental off‐target side effects.

## 3 Conclusions

In this work, we report a potent multifunctional nanoplatform allowing synergistic chemo‐photothermal therapy of tumors, guided by both ultrasound and photoacoustic imaging. This has been achieved by incorporating PFP and ICG into biodegradable hollow mesoporous organosilica nanoparticles. The pores in the HMONs were then gated with a PTX prodrug, and the resultant composite subjected to PEGylation. The resultant ICG/PFP@HMOP‐PEG formulation showed drug release which was accelerated in reducing environments typical of cancer cells. This leads to effective intracellular ICG delivery. The nanoparticles cause NIR‐responsive hyperthermia, permitting both photothermal therapy and photoacoustic imaging. They can additionally induce a liquid−gas phase transformation of PFP, resulting in effective ultrasound imaging. The intrinsic NIR fluorescence imaging ability of ICG/PFP@HMOP‐PEG permits real‐time visualization of its distribution in vivo, which is conducive to both accurate diagnosis and treatment. In vitro and in vivo evaluations confirm that ICG/PFP@HMOP‐PEG leads to potent and synergistic chemo‐photothermal therapy. This work thus provides an efficient strategy for successful theranostics, particularly for enhanced dual‐modality imaging guided photothermal therapy of cancer.

## 4 Experimental Section

All animal studies were supervised by the Laboratory Animal Center of Shanghai General Hospital, and procedures carried out in accordance with protocols approved by the Animal Care and Use Committee at Shanghai General Hospital. Detailed experimental materials and methods can be found in the Supporting Information.

## Conflict of Interest

The authors declare no conflict of interest.

## Supporting information

Supplementary
