# Supplementary material for: A Multifunctional Biodegradable Nanocomposite for Cancer Theranostics
Source: Adv Sci (Weinh). 2019 May 21;6(14):1802001. doi: 10.1002/advs.201802001 (PMC6661946; doi:10.1002/advs.201802001)
Supplement: Supplementary file 1 — Supplementary [file ADVS-6-1802001-s001.pdf]

Copyright WILEY-VCH Verlag GmbH & Co. KGaA, 69469 Weinheim, Germany, 2019.

Supporting Information

for *Adv. Sci.,* DOI: 10.1002/advs.201802001

A Multifunctional Biodegradable Nanocomposite for Cancer Theranostics

Jianrong Wu, Gareth R. Williams, Shiwei Niu, Feng Gao, Ranran Tang, and Li-Min Zhu**

Supporting Information

A Multifunctional Biodegradable Nanocomposite for Cancer Theranostics

Jianrong Wu,[a] Gareth R. Williams,[b] Shiwei Niu,[a] Feng Gao,[c] Ranran Tang,[d,] and Li-min Zhu[a,]**

Dr. J. Wu, Dr. S. Niu, Prof. L. Zhu

College of Chemistry, Chemical Engineering and Biotechnology, Donghua University, Shanghai 201620, P.R. China

Email: lzhu@dhu.edu.cn

Dr. G. R. Williams

UCL School of Pharmacy, University College London

29-39 Brunswick Square, London WC1N 1AX, UK

Dr. F. Gao

Department of Ultrasound, Shanghai General Hospital, Shanghai Jiaotong University School of Medicine, Shanghai 200080, P. R. China

Dr. R. Tang

Women's Hospital of Nanjing Medical University, Nanjing Maternity and Child Health Care Hospital, Nanjing, 210004, China.

***Corresponding authors**: lzhu@dhu.edu.cn (L-M. Zhu) 13190186401@163.com (R. Tang).

Experimental Section

Materials: Tetraethylorthosilicate (TEOS), triethanolamine (TEA), N,N'-dicyclohexylcarbodiimide (DCC), N-hydroxysuccinimide (NHS), dichloromethane (DCM), diethyl ether and ammonia solution were procured from the Sinopharm Chemical Reagent Co. (Shanghai, China). Indocyanine green (ICG), bis[3-(triethoxysilyl)propyl]tetrasulfide (BTES), 3-aminopropyltriethoxysilane (APTES), 4-dimethylaminopyridine (DMAP), and 3,3′-dithiodipropionic acid (DPA) were purchased from the Aladdin Reagent Co. Ltd. (Shanghai, China). Paclitaxel (PTX) and 4,6-diamino-2-phenylindole (DAPI) was provided by the Dalian Meilun Biotech Co., Ltd. (Dalian, China). Perfluoropentane (PFP) was supplied by the Energy Chemical Co., Ltd. (Shanghai, China). Glutathione (GSH), cetyltrimethyl ammonium chloride (CTAC), calcein AM, and propidium iodide (PI) were obtained from Sigma-Aldrich (St Louis, MO, USA). Methoxypolyethylene glycol acid (mPEG-COOH, Mw: 3000 Da) was received from the Shanghai Yanyi Biotechnology Corporation (Shanghai, China). A cell counting kit-8 (CCK-8) was purchased from the Beyotime Institute of Biotechnology (Shanghai, China). Fetal bovine serum (FBS), penicillin-streptomycin solution, 0.05% trypsin-EDTA, phosphate buffered saline (PBS), and Dulbecco's modified Eagle medium (DMEM) were sourced from Thermo Scientific (Beijing, China). A human breast cancer cell line (MDA-MB-231) was provided by KeyGEN Bio TECH Co., Ltd (Nanjing, China). All chemicals were used as received without further purification.

Synthesis of HMONs-NH$_2$: Hollow mesoporous organosilica nanoparticles were synthesized by an ammonia-assisted selective etching strategy. 6 g of CTAC was mixed with 225 μL of TEA, dissolved in 100 mL of anhydrous ethanol, and stirred for 20 min at room temperature. TEOS (0.5 mL) was added dropwise and the resultant mixture stirred at 80 °C for 1 h to obtain SiO_2. Next, core-shell SiO_2@MONs nanoparticles were prepared by adding a mixture of TEOS (0.75 mL) and BTES (0.6 mL) and stirring for another 4 h at 80 °C, followed by centrifugation and washing with ethanol three times. To remove the template CTAC, the product was re-dispersed in 80 mL of HCl in MeOH (10%, v/v) and refluxed at 80 °C for 8 h. This process was repeated three times and resulted in the generation of SiO_2@MONs. 0.5 mL of ammonia solution was added into the SiO_2@MON suspension, and allowed to react for 3 h at 95 °C. Finally, the resultant HMONs were recovered after washing several times with water and methanol, and drying under vacuum. Thereafter, the amination process was conducted by reacting 500 mg of HMONs with 1.5 mL of APTES in 20 mL of methanol under reflux for 24 h at 80 ºC. The HMONs-NH_2 product was centrifuged and washed with ethanol three times, then dried at 60 °C under high vacuum overnight. Meanwhile, *in vitro* degradation experiments were carried out in simulate body fluid (SBF) with or without 10 mM glutathione (GSH) according to protocols described in the literature. [1]

Synthesis of PTX-DPA (PTX-SS-COOH) Prodrug: DPA (0.1 mmol) was dissolved in 5 mL of anhydrous DCM, and NHS (0.4 mmol) was introduced under stirring. Subsequently, DCC (0.2 mmol) was added. After stirring for 24 h at room temperature

under N_2, the mixture was filtered to remove N,N-dicyclohexylurea. Thereafter, PTX (0.2 mmol) was dissolved in anhydrous DCM and the activated DPA solution was added dropwise under stirring. DMAP (0.2 mmol) was added and the resultant solution stirred at room temperature for 2 days under N_2. The reaction solution was extracted, washed with 1% HCl (40 mL) and dried with anhydrous Na_2SO_4. After concentration to roughly 1 mL under vacuum, the desired product was precipitated by the addition of 10 mL of cold diethyl ether and dried under vacuum to obtain PTX-SS-COOH (yield = 84.6%).

Synthesis of ICG/PFP@HMOP-PEG: For PFP loading, 50 mg of HMONs-NH_2 was placed in an Eppendorf tube. A vacuum was applied to remove the air in the tube, and 200 μL of PFP was injected, followed by ultrasound sonication for 2 min in an ice bath to produce PFP-loaded HMONs (PFP@HMONs). The product was re-dispersed in PBS (25 mL). Subsequently, ICG loading was carried out by adding 10 mL of PBS containing 20 mg of ICG to the above solution and shaking for 24 h in the dark. The ICG/PFP@HMONs produced were collected by centrifugation. The loading capacity was calculated by measuring the change in concentration of ICG during the loading process. The loading content of HMONs was calculated as follows:

Loading content (%) = 100 × (weight of ICG loaded into the HMONs) / (weight of the ICG/PFP@HMONs).

For PTX prodrug capping, PTX-SS-COOH (30 mg) was dispersed in 50 mL of water and EDC (10.5 mg) and NHS (6.3 mg) were added. After vigorous stirring for 2 h in the dark, 40 mL of water containing 50 mg of ICG/PFP@HMONs was added

under gentle magnetic stirring. 12 h later the reaction was complete, giving ICG/PFP@HMOP. PEGylation was achieved by adding mPEG-COOH during the preparation of ICG/PFP@HMOP to obtain ICG/PFP@HMOP-PEG.

Characterization: The morphology of the nanoparticles was characterized using high-resolution transmission electron microscopy (TEM, Talos F200S, FEI, Rotterdam, Netherlands) equipped with an energy dispersive spectroscopy (EDS) attachment. N_2 adsorption/desorption isotherms were obtained by an absorption analyzer (Micromeritics Instruments Corporation, Atlanta, GA, USA), with the specific surface area and pore size determined using the BET and BJH methods respectively. Size and ζ-potential were quantified on a Zetasizer Nano ZS90 (Malvern Instruments Ltd., Malvern, UK). ^{1}H NMR spectra were recorded on a Unity Inova 400 spectrometer (Agilent, Santa Clara, CA, USA). Solid state ^{29}Si and ^{13}C CP/MAS NMR spectra were collected using an AVANCE III HD 500 MHz NMR spectrometer (Bruker, Zurich, Switzerland). FT-IR spectra were obtained with the aid of an Prestige-21 spectrometer (Shimadzu, Kyoto, Japan). UV-vis-NIR absorption spectra were recorded on a UV-3600 spectrophotometer (Shimadzu, Kyoto, Japan). Photothermal properties were studied by using an 808 nm near-infrared (NIR) laser (Shanghai Xilong Optoelectronics Technology Co. Ltd., Shanghai, China) and an infrared thermal camera (FLIR A300, Pumeng Technology, Shanghai, China).

Photothermal Effects and In Vitro Drug Release: To evaluate the photothermal effect of ICG/PFP@HMOP-PEG, a 0.5 mL dispersion at different ICG concentrations (2.5, 5, 10, 20 μg/mL) was irradiated under an NIR laser (808 nm, 1.0 W/cm^2) for 5

min. In a second set of experiments, a given ICG concentration (5 μg/mL) was irradiated under different power densities of the NIR laser. The temperature change of the samples was monitored with a thermal infrared imaging camera (FLIR A300, Pumeng Technology, Shanghai, China). Free ICG (5 μg/mL) was used as a control for cycling experiments.

In vitro ICG release from ICG/PFP@HMOP-PEG was studied under different pH conditions (7.4 and 5.5), with or without 10 mM GSH. Briefly, 5 mg of ICG/PFP@HMOP-PEG were dispersed in PBS (2 mL). 1.0 mL was transferred into a dialysis bag (MWCO: 5000 Da) and dialyzed against PBS. At designated time intervals, the amount of ICG in the dialysate was analyzed with a RF-530/PC spectrofluorophotometer (Shimadzu, Kyoto, Japan) at λ_{ex} = 780 nm, λ_{em} = 850 nm. Drug release from the nanoparticles was measured using the same dialysis method, but PTX was quantified with high performance liquid chromatography (HPLC; Agilent 1260, Agilent Technologies Inc, Cotati, CA). The mobile phase was a mixture of acetonitrile and water (60:40, v/v) and the flow rate was set at 1.0 mL/min. All experiments were repeated three times.

Measurement of Bubble Release from ICG/PFP@HMOP-PEG: 0.5 mL of a ICG/PFP@HMOP-PEG suspension (6 mg/mL in PBS, with or without 10 mM GSH) was placed onto a glass slide and covered with a cover slip. The samples were exposed to NIR laser irradiation (1.0 W/cm^2) for 5 min. The glass slide was then fixed with 4% paraformaldehyde and imaged under a confocal laser scanning microscope (CLSM, FV1000, Olympus, Tokyo, Japan).

In Vitro US/PA Imaging: ICG/PFP@HMOP-PEG was dispersed in water at two different concentrations (5 and 20 mg/mL). Before and after NIR irradiation, static contrast ultrasound images were collected under B fundamental imaging (BFI) and contrast mode on a IU-Elite US imaging system (Philips, Eindhoven, the Netherlands) with a mechanical index at 0.07 and a frequency of 40 MHz. For *in vitro* PA imaging, ICG/PFP@HMOP-PEG dispersions at varied ICG concentrations (2, 5, 10, and 20 μg/mL) were injected into individual holes on a home-made agar plate and PA images obtained using a Vevo LAZR PA imaging system (Visualsonics Inc., Toronto, Canada). The excitation wavelength was set from 700 to 950 nm with a 10 nm interval, and regions of interest were fixed at 20 mm.

In Vitro Bubble-Enhanced Cellular Uptake: The cellular uptake of the HMON nanoparticles was studied using flow cytometry and CLSM. For CLSM, MDA-MB-231 cells were seeded at a density of 5×10^4 cells/well (in 1 mL) in a confocal culture dish, with the culture medium comprising DMEM supplemented with 1% v/v penicillin-streptomycin solution and 10% v/v fetal bovine serum (FBS). The plate was incubated in a 5% CO_2 air atmosphere for 24 h at 37 °C. After 24 h, the medium was aspirated and replaced with fresh medium containing FITC-ICG@HMOP-PEG or FITC-ICG/PFP@HMOP-PEG at a given ICG concentration, and incubated for another 4 h at room temperature. For NIR laser irradiation experiments, cells were irradiated with an 808 nm laser of 1.0 W/cm^2 power density for 5 min after 2 h incubation. After the 4 h incubation time, the cells were washed with PBS and fixed with 4% paraformaldehyde for 0.5 h. Finally, they

were stained with DAPI and observed using CLSM (LSM 780, Carl Zeiss, Germany) using the fluorescence of FITC to assess uptake.

Quantitative analyses of the intracellular FITC fluorescence were undertaken by flow cytometry (FACS Aria TM III, BD Biosciences, San Jose, CA, USA). The uptake of the nanoparticles was further studied by bio-TEM, using the same procedure as described in a previous report[2] and a JEM-1230 microscope (JEOL, Tokyo, Japan).

In Vitro Cytotoxicity Assays: MDA-MB-231 cells in DMEM supplemented with FBS and penicillin/streptomycin were seeded in 96-well plates (2×10^4 cells/well, 200 μL) and incubated for 24 h. The culture medium was then replaced with DMEM solutions of free PTX or suspensions of ICG/PFP@HMOP-PEG nanoparticles at different concentrations. After 24 h incubation, relative cell viabilities were determined using the standard CCK-8 proliferation assay, in accordance with the manufacturer's instructions. To assess chemo-photothermal therapeutic efficacy, cells (seeded as above) were exposed to different treatments, including free ICG, ICG/PFP@HMONs-PEG and ICG/PFP@HMOP-PEG at a fixed concentration of ICG (5 μg mL^{-1}), with or without a 5 min exposure to 808 nm NIR light irradiation (1.0 W/cm^2). After additional 24 h incubation at 37 °C, the CCK-8 assay was performed.

To evaluate apoptosis *in vitro*, MDA-MB-231 cells were seeded in 6-well plates at a density of 5×10^4 cells/well (in 2 mL DMEM) and cultured overnight. Cells were next incubated with free PTX (3.5 μg mL^{-1}), free ICG, or ICG/PFP@HMOP-PEG at equivalent ICG concentrations of 5 μg mL^{-1} for another 12 h, with or without laser

irradiation for 5 min after 2 h of incubation. Afterwards, the cells were rinsed thoroughly with PBS, and trypsin-EDTA (100 μL) added to resuspend them. The cells were stained in a glutaraldehyde binding buffer containing PI and Annexin-V-FITC for 15 min, and then analyzed by flow cytometry. To visually demonstrate the cytotoxicity of ICG/PFP@HMOP-PEG, MDA-MB-231 cells which had been subjected to the different treatments were co-stained with calcein AM (5 μg mL^{-1}) and PI (10 μg mL^{-1}) and then imaged using a digital microscope (Leica TCS SP8, Leica Microsystems, Wetzlar, Germany).

To examine the apoptotic profile of various treatments on tumor cells, a molecular level evaluation of apoptosis was performed using the real-time reverse transcriptase polymerase chain reaction (RT-qPCR). MDA-MB-231 cells (1×10^6 cells/mL) were harvested after being incubated with free PTX (3.5 μg mL^{-1}), free ICG, or ICG/PFP@HMOP-PEG at equivalent ICG concentrations of 5 μg mL^{-1} for 12 h, with or without laser irradiation for 5 min after 2 h of incubation. Several specific biological bio-markers for the apoptotic cell death including two representative pro-apoptotic genes (*Bax*, *Caspase-3*) and one anti-apoptotic gene (*Bcl-2*) were selected for determination at the messenger RNA (mRNA) level. The total RNA was isolated from the cells using the Trizol reagent (Invitrogen, Carlsbad, CA, USA) according to the manufacturer's instructions. 1 μL of each cDNA and 1 μg of total RNA from each sample was used for reverse transcription in a total volume of 20 μL. PCR cycling conditions were: 40 cycles of 94 °C for 1 min, 60 °C for 1 min, and 72 °C for 2 min. The primers used for PCR were: *Bcl-2* forward primer 5'-CTTTGAGTTCGGTGGGGTCA-3' and reverse 5'-GGGCCGTACAGTTCCACAAA -3', product length 162 bps; *Bax* forward primer 5'-TCATGGGCTGGACATTGGAC-3' and reverse 5'-GAGACAGGGACATCAGTCGC-3', product length 114 bps; *Caspase-3* forward primer 5'-AGCTTGGAACGGTACGCTAA-3' and reverse 5'-

CCACTGACTTGCTCCCATGT-3’, product length 113 bps; and *β-actin* forward primer 5’-TGAGCTGCGTTTTACACCCT-3’, and reverse 5’-GCCTTCACCGTTCCAGTTTT-3’, product length 198 bps (Tm 60 ℃). The fluorescence signal was determined at the end of each cycle, and the results analyzed using the $2^{-\triangle\triangle CT}$ method with β-actin as an internal reference.

Animals and Tumor Model: 4-6-week old female BALB/c nude mice (18−20 g) and Sprague-Dawley (SD) rat (180-220 g) were obtained from the Shanghai Laboratory Animal Center (Shanghai, China). All animal studies were supervised by the Laboratory Animal Center of Shanghai General Hospital, and procedures carried out in accordance with protocols approved by the Animal Care and Use Committee at Shanghai General Hospital. To establish the tumor model, MDA-MB-231 cells (2×10^6/mouse, 100 μL) were injected subcutaneously into one hind leg of each mouse. The tumor size and body weight were monitored every 2 days post-treatment, and tumor volumes were calculated as $V = [(\text{length}) \times (\text{width})^2]/2$. *In vivo* experiments were begun when the tumor volume reached approximately 100 mm^3.

In Vivo Multimodal Imaging: For US imaging, 100 μL of a ICG/PFP@HMOP-PEG suspension (3 mg/mL in terms of ICG) was intravenously injected into a MDA-MB-231 tumor-bearing mouse. After 24 h, B-mode ultrasound imaging was conducted on a Philips IU-Elite US imaging system (Eindhoven, Netherlands) with a mechanical index at 0.07 and a frequency at 40 MHz before and after 808 nm NIR laser irradiation (1 W cm^{-2}, 5 min). PA imaging was also performed using a Vevo LAZR PA Imaging System (Visualsonics Inc., Toronto, Canada) at different time points (pre-injection, 1, 2, 12, 24 and 48 h). The excitation wavelength was set from

700 to 950 nm and regions of interest were fixed at 20 mm.

For *in vivo* fluorescence imaging, MDA-MB-231 tumor-bearing mice were randomly divided into three groups (n = 3 per group) and given an intravenous injection of 200 μL of free ICG or ICG-based nanoparticles (3 mg/mL in terms of ICG) via the tail vein. The mice were imaged at different time intervals (1, 2, 4, 8, 12, and 24 h post-injection) on a Lumina III in vivo imaging system (PerkinElmer, Waltham, MA, USA). An 831 nm filter was used to measure the fluorescence emission of ICG (λ_{ex}= 780 nm).

In Vivo Biodistribution and Pharmacokinetics: MDA-MB-231 tumor-bearing mice (n=3) were administrated with ICG/PFP@HMOP-PEG, ICG/PFP@HMOP or free ICG (5 mg ICG equiv./kg). 24 h post-injection, the major organs (liver, spleen, kidney, heart, lung) and tumor were collected for *ex vivo* fluorescence imaging using the Lumina III system detailed above. The ICG fluorescence intensity was also quantified following homogenization of the organs.

In vivo pharmacokinetics were investigated in SD rats (n=3 per group) intravenously injected with ICG/PFP@HMOP-PEG, ICG/PFP@HMOP or free ICG at an ICG dosage of 5 mg/kg. ~20 μL of blood was taken at different time points post-injection and dispersed in lysis buffer before the ICG concentration of the sample was determined by fluorescence measurements (RF-530/PC instrument, Shimadzu, Kyoto, Japan). The excitation and emission wavelengths were set at 780 and 850 nm, respectively.

In Vivo Antitumor Activity and Biosafety: Tumor-bearing mice were randomly

divided into six groups (5 animals per group) and treated with (1) saline, (2) free PTX, (3) ICG/PFP@HMOP-PEG, (4) free ICG and NIR, (5) ICG@HMOP-PEG+NIR and (6) ICG/PFP@HMOP-PEG+NIR. The doses of ICG, PTX and HMONs were 5 mg/kg, 4 mg/kg, and 9.5 mg/kg, respectively. For the NIR groups, the tumor regions were subjected to NIR irradiation (1.0 W/cm^2, 10 min) at 8 h post-injection. The temperature change of the tumor sites was carefully monitored with an infrared thermal camera (FLIR A300, Pumeng Technology, Shanghai, China). The tumor size and body weight were measured every 2 days after treatment. To further evaluate the antitumor activity, a histology study was also carried out. The tumor tissues from each group were dissected, stained with hematoxylin and eosin (H&E), TdT-mediated dUTP Nick-End Labeling (TUNEL) and Ki-67 and observed by optical microscopy.

For *in vivo* biosafety evaluation, one mouse from each group was sacrificed on the 3rd day after treatment. The main organs (heart, liver, spleen, lungs, kidneys) were collected for H&E staining. Meanwhile, healthy female Balb/c mice (n = 4) were injected with 200 μL of ICG/PFP@HMOP-PEG (10 mg/kg). Mice injected with PBS (n=4) were used as a control. 0.5 mL of blood from each mouse was collected 15 days post-injection for blood and biochemistry tests, and analyzed according to the protocols described in previous work.[3]

Statistical Analysis: All experimental data are presented as mean ± S.D. Each experiment was performed at least in triplicate, and data analyzed with the SPSS software using one-way ANOVA followed by a post hoc Tukey’s test.

References

[1] P. Huang, Y. Chen, H. Lin, L. Yu, L. Zhang, L. Wang, Y. Zhu, J. Shi, *Biomaterials* **2017,** *125*, 23.

[2] C. Li, Y. Zhang, Z. Li, E. Mei, J. Lin, F. Li, C. Chen, X. Qing, L. Hou, L. Xiong, H. Hao, Y. Yang, P. Huang, *Adv. Mater.* **2018,** *30*, 1706150.

[3] Y. Zheng, L. Wang, T.M. Krupka, Z. Wang, G. Lu, P. Zhang, G. Zuo, P. Li, H. Ran, H. Jian, *Eur. J. Radiol.* **2013,** *82*, 512.

Supplementary Figures and Tables

Table S1. Pharmacokinetic parameters of ICG after intravenous injection of the different formulations (n = 4).

Parameter	Free ICG	ICG/PFP@HMONs	ICG/PFP@HMON-PEG
C_{max} (iD%/g)	33.6 ± 1.8	49.7 ± 2.5	65.2± 2.7
$AUC_{0-\infty}$ (μg/mL•h)	19.3 ± 6.7	91.2 ± 11.3	152.1 ± 14.8
MRT (h)	1.4 ± 0.78	13.3 ± 1.6	15.4 ± 1.9

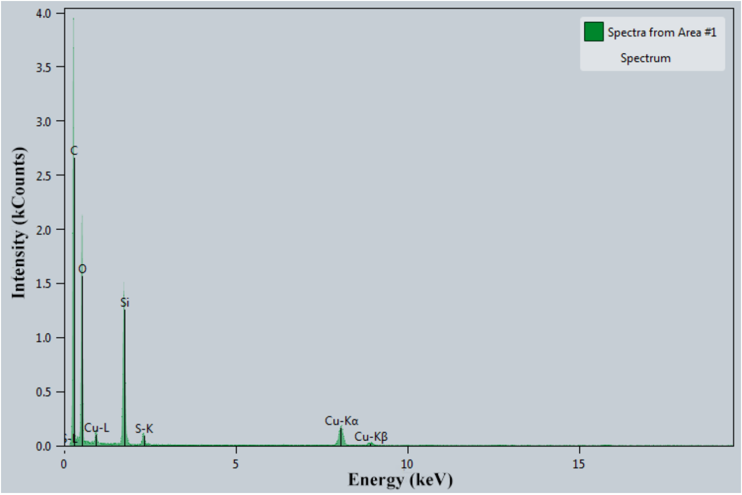

Figure S1. EDX spectrum of the HMONs prepared in this work.

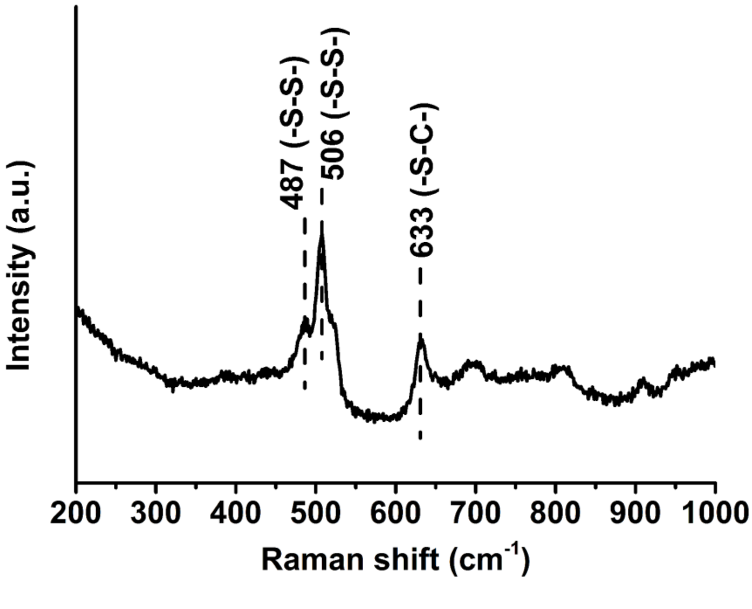

Figure S2. Raman spectrum of the HMONs.

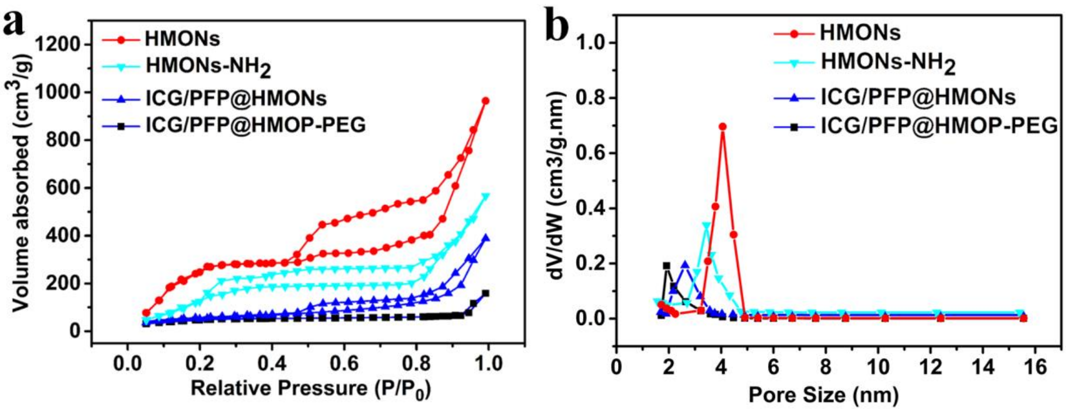

Figure S3. (a) N_2 adsorption-desorption isotherms and (b) the corresponding pore-size distribution for HMONs, HMONs-NH_2, ICG/PFP@HMONs, and ICG/PFP@HMOP-PEG.

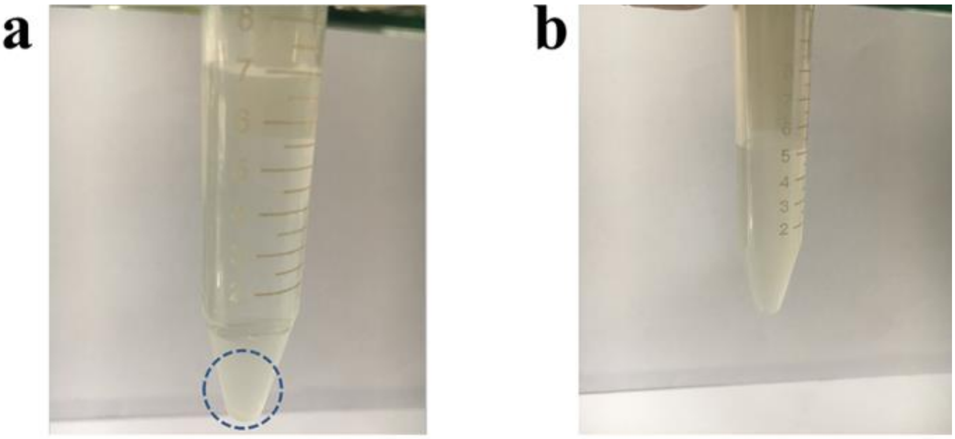

Figure S4. Photographs of (a) free PFP and (b) PFP@HMONs in PBS.

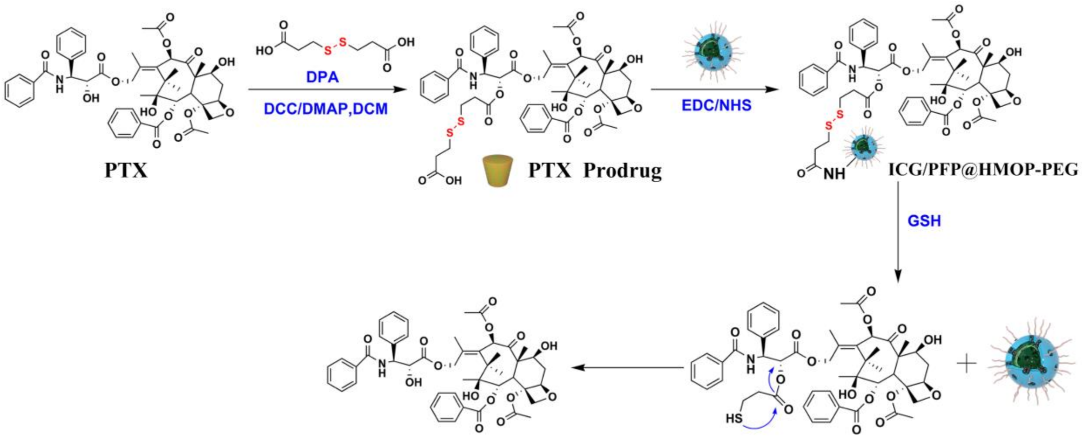

Figure S5. The synthetic route to prepare the PTX prodrug and ICG/PFP@HMOP-PEG nanoplatform, and the mechanism of GSH-responsive drug release.

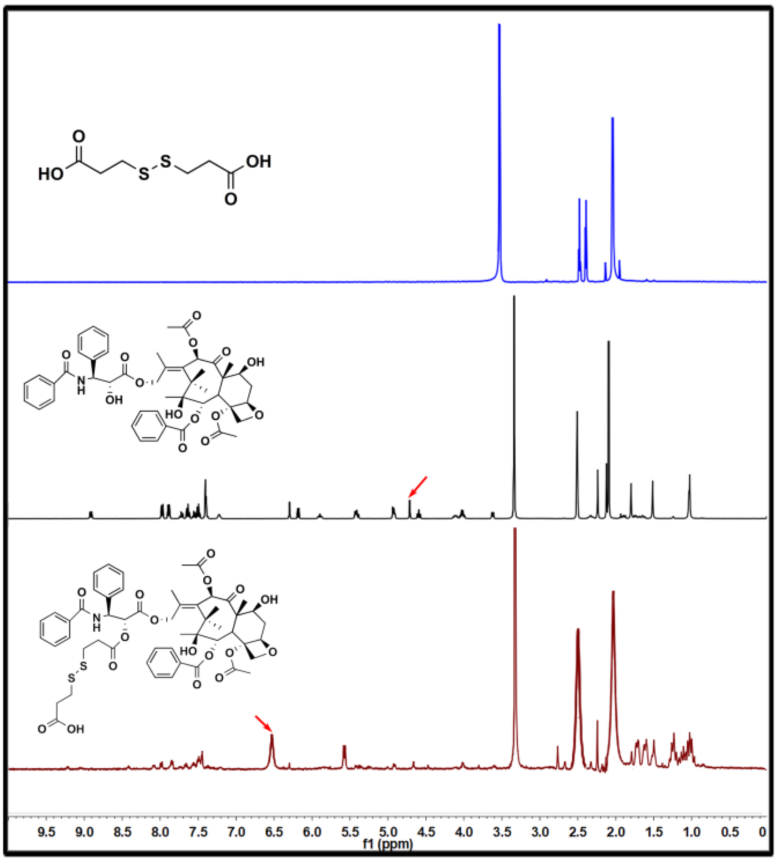

Figure S6. ^{1}H NMR spectra of DPA in $CDCl_3$ (top), PTX in DMSO-d6 (middle), and PTX-SS-COOH in DMSO-d6 (bottom).

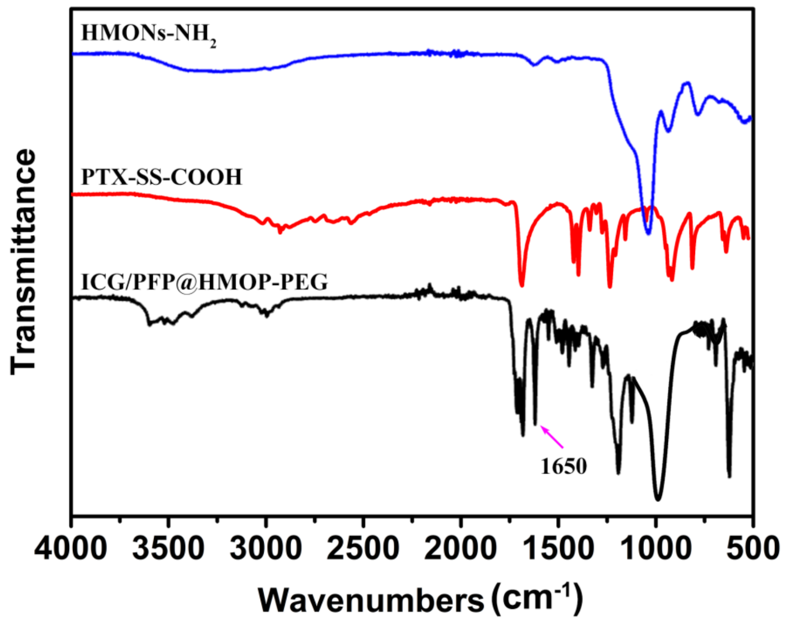

Figure S7. FT-IR spectra of HMONs-NH_2, PTX-SS-COOH, and ICG/PFP@HMOP-PEG.

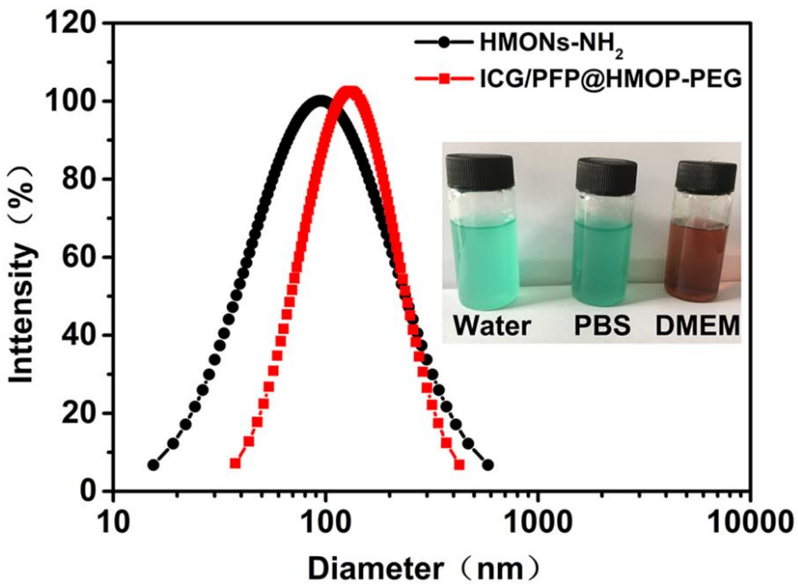

Figure S8. Hydrodynamic diameters of HMONs-NH_2 and the ICG/PFP@HMOP-PEG nanoparticles. Inset: photographs of the ICG/PFP@HMOP-PEG nanoparticles suspended in water, PBS, and DMEM.

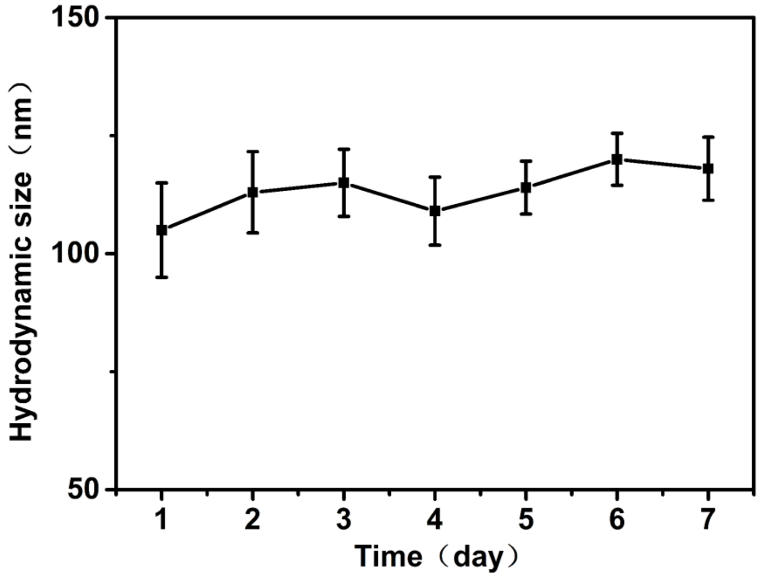

Figure S9. The hydrodynamic size of ICG/PFP@HMOP-PEG dispersed in PBS for 7 days.

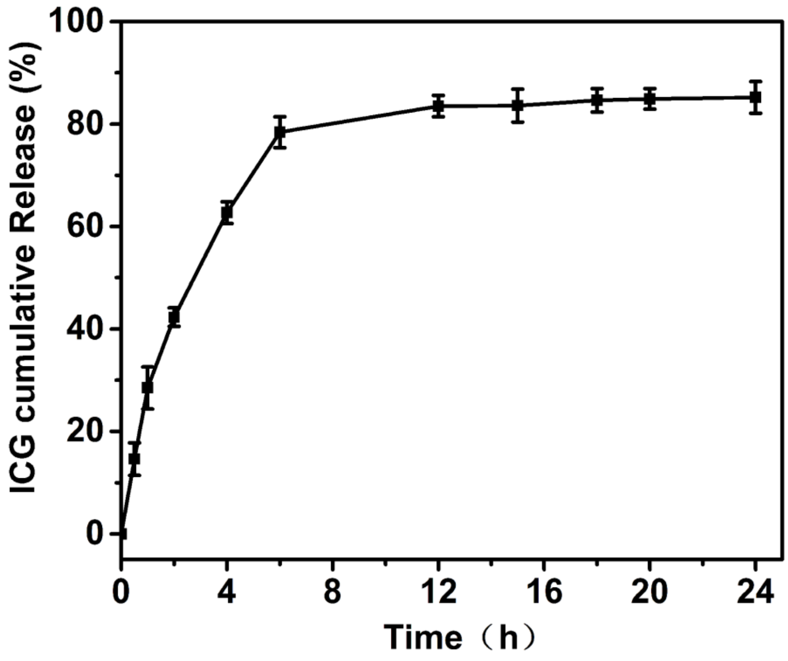

Figure S10. In vitro ICG release from ICG/PFP@HMONs-PEG at pH 7.4.

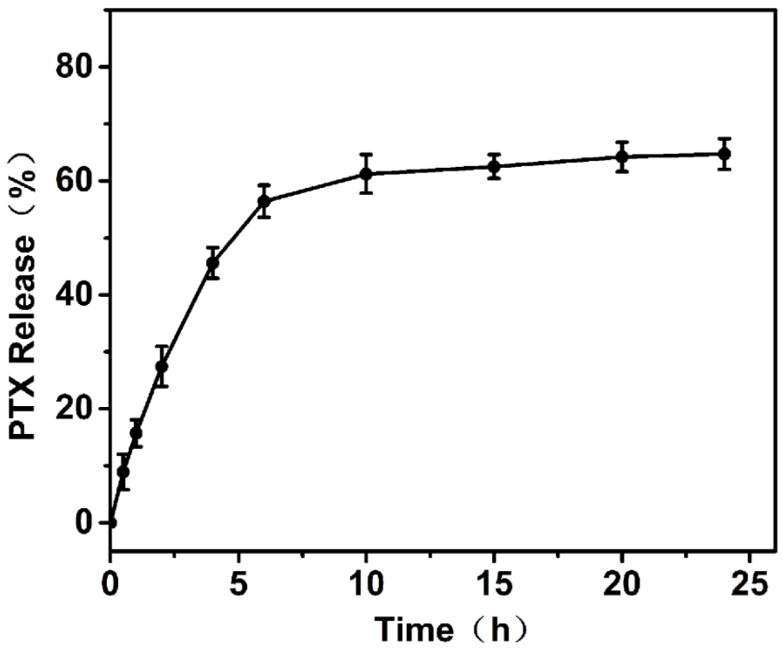

Figure S11. In vitro PTX release from ICG/PFP@HMONs-PEG at pH 5.5 in the presence of 10 mM GSH.

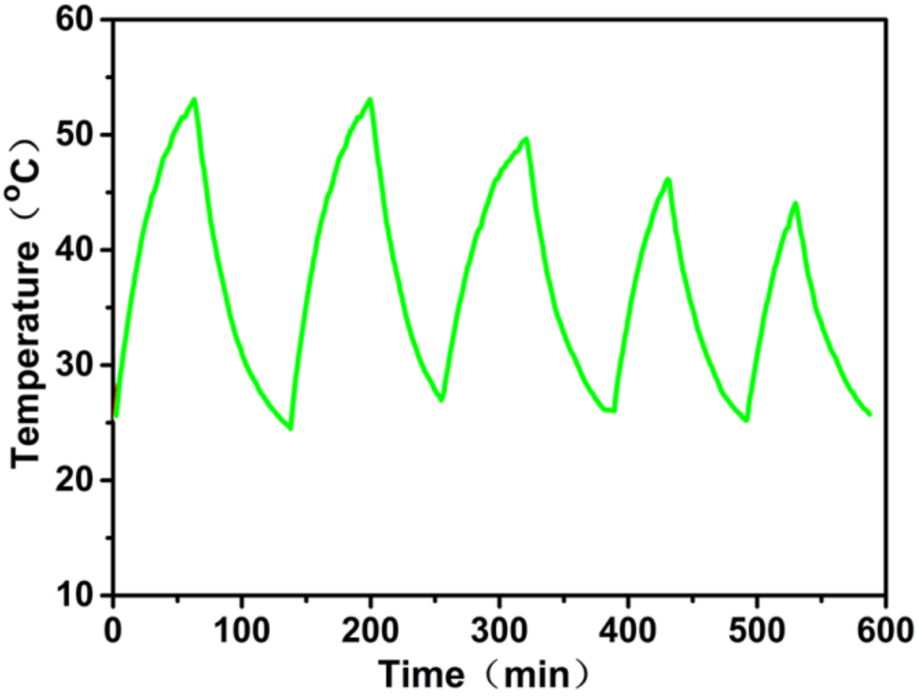

Figure S12. Photothermal stability of free ICG over five cycles of laser NIR irradiation.

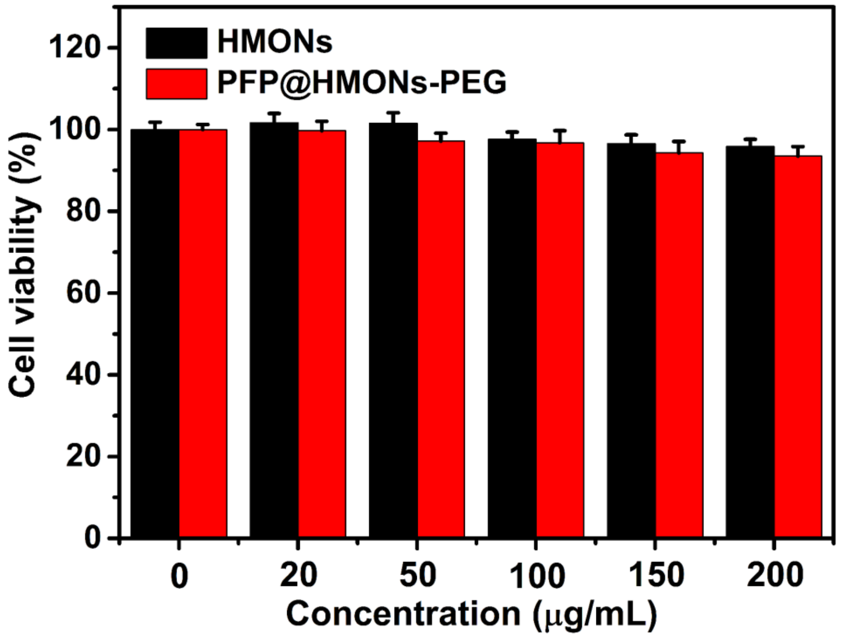

Figure S13. Viability of MDA-MB-231 cells after incubation with HMONs and PFP@HMONs-PEG for 24 h.

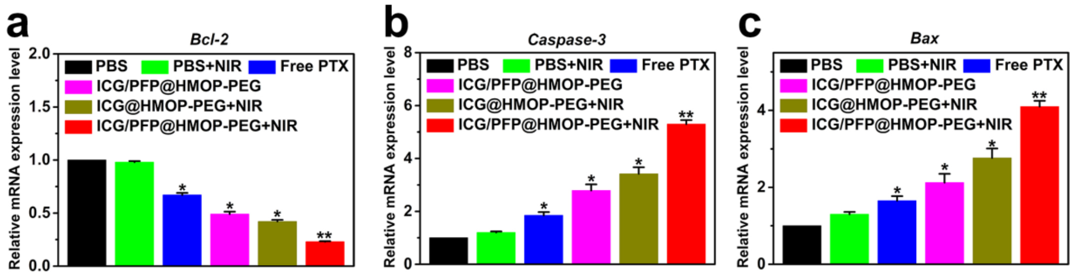

Figure S14. Relative mRNA expression levels of (a) *Bcl-2*, (b) *Caspase-3*, and (c) *Bax* in MDA-MB 231 cells. Data are expressed as mean ± SD (n = 5), *P < 0.05, **P < 0.01.

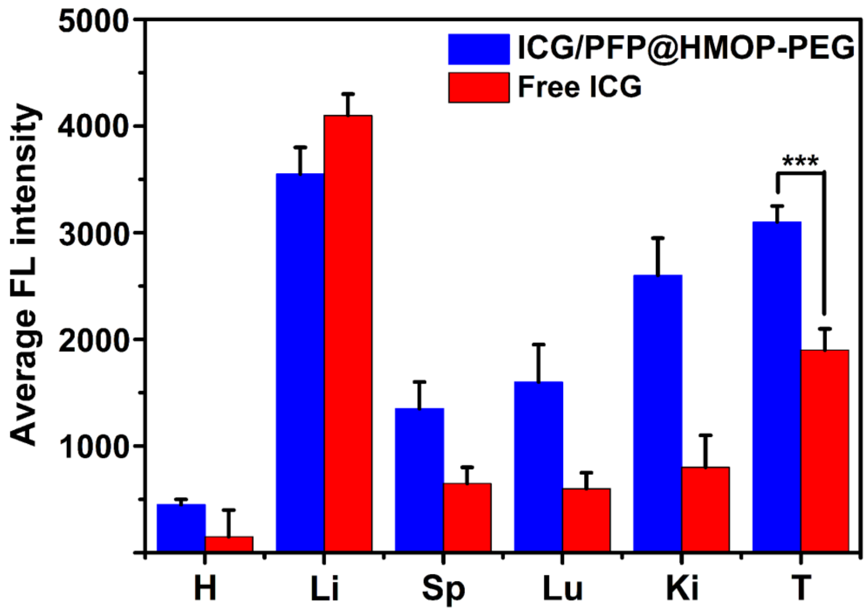

Figure S15. Quantification of *ex vivo* fluorescence intensities for the different organs depicted in Figure 6g. H, Li, Sp, Lu, Ki, and T denote the heart, liver, spleen, lung, kidney, and tumor, respectively.

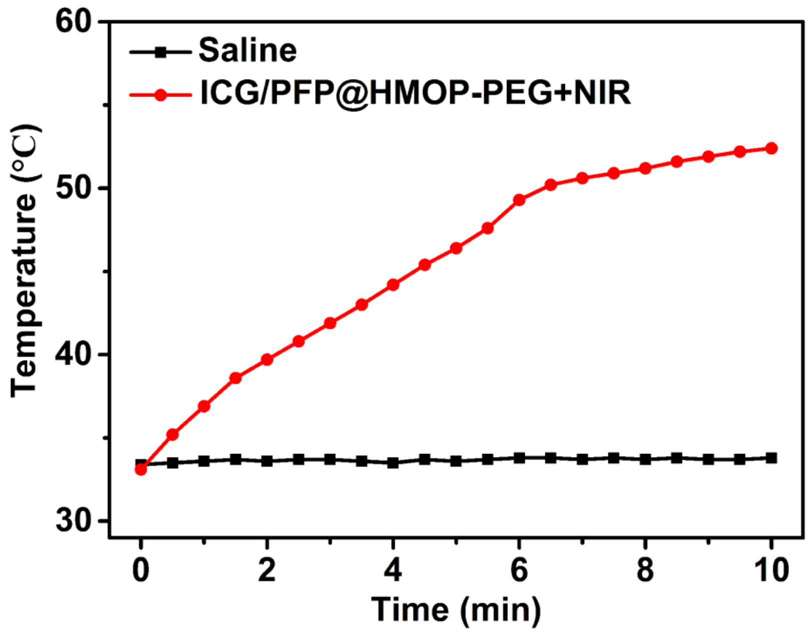

Figure S16. Temperature changes of the tumor in mice *i.v.* injected with saline or ICG/PFP@HMOP-PEG and exposed to 808 nm laser irradiation.

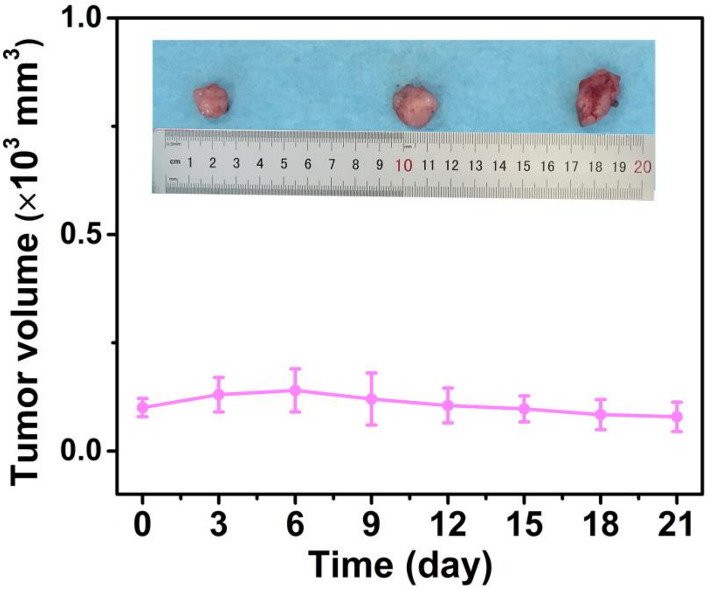

Figure S17. The tumor volume and photographs of the tumors excised from the PFP@HMOP-PEG+NIR group.

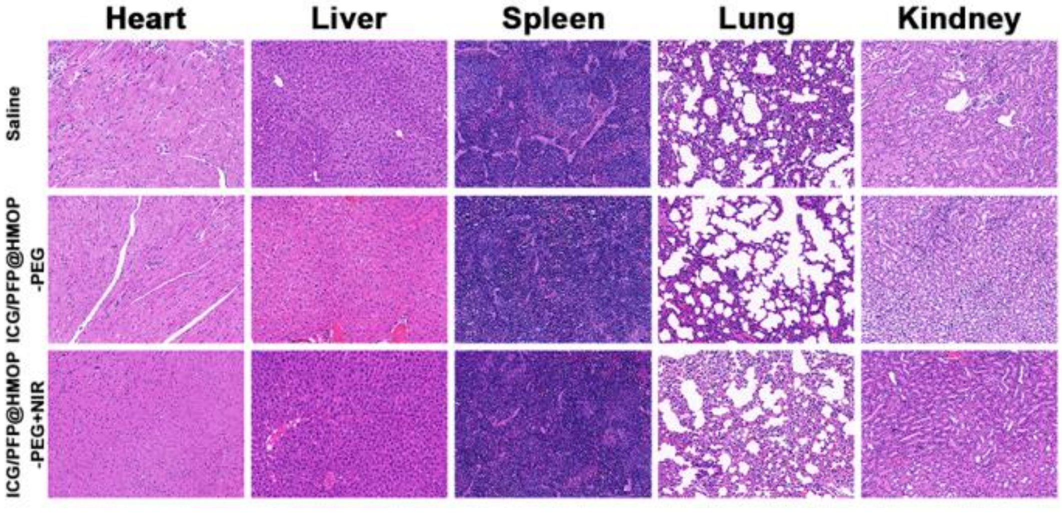

Figure S18. Representative H&E stained images of the major organs, collected on day 21.

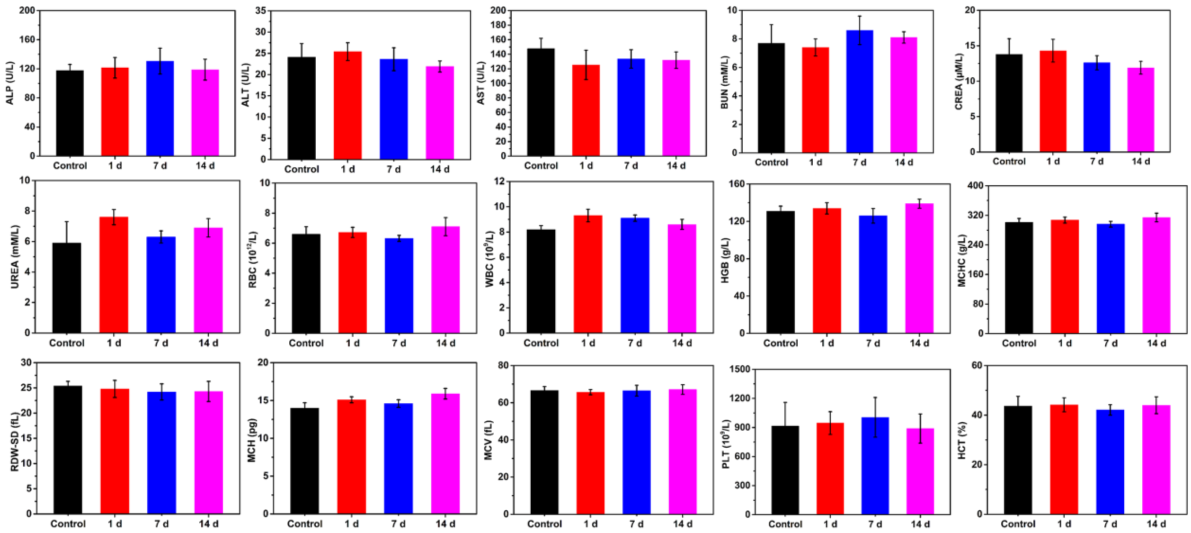

Figure S19. Blood biochemistry and hematology data for healthy mice treated with saline, and with ICG/PFP@HMOP-PEG and NIR laser irradiation (data are shown for day 1, 7, and 28).
